# Patient perceptions of health-related quality of life in giant cell arteritis: international development of a disease-specific patient-reported outcome measure

**DOI:** 10.1093/rheumatology/keab076

**Published:** 2021-02-02

**Authors:** Joanna C Robson, Celia Almeida, Jill Dawson, Alison Bromhead, Emma Dures, Catherine Guly, Elizabeth Hoon, Sarah Mackie, Mwidimi Ndosi, John Pauling, Catherine Hill

**Affiliations:** 1 Centre for Health and Clinical Research, University of the West of England; 2 Bristol Royal Infirmary, University Hospitals and Weston Bristol NHS Foundation Trust, Bristol; 3 Nuffield Department of Population Health (HSRU), University of Oxford, Oxford, UK; 4 School of Public Health, University of Adelaide, Adelaide, SA, Australia; 5 Leeds Institute of Rheumatic and Musculoskeletal Medicine, NIHR (National Institute for Health Research) Leeds Biomedical Research Centre, University of Leeds; 6 Leeds Teaching Hospitals NHS (National Health Service) Trust, Leeds; 7 Department of Rheumatology, Royal National Hospital for Rheumatic Diseases; 8 Department of Pharmacy & Pharmacology, University of Bath, Bath, UK; 9 Rheumatology Unit, The Queen Elizabeth Hospital, Woodville; 10 Rheumatology Unit, The Royal Adelaide Hospital, Adelaide, SA, Australia

**Keywords:** giant cell arteritis, quality of life, patient reported outcome

## Abstract

**Objectives:**

GCA is a large vessel vasculitis (LVV) presenting with headache, jaw claudication, musculoskeletal and visual involvement. Current treatment is glucocorticoids and anti-IL-6 tocilizumab in refractory disease. The objective of this study was to explore the impact of GCA and its treatment on people’s health-related quality of life (HRQoL), to inform the development of a disease-specific patient-reported outcome measure (PROM) for use in clinical trials and practice.

**Methods:**

Participants from the UK and Australia, with biopsy- or imaging-confirmed GCA, were interviewed to identify salient aspects of HRQoL in relation to GCA and its treatment. Purposive sampling included a range of demographic and disease features (cranial, LVV-GCA and visual involvement). Inductive analysis identified individual themes of importance, then domains. Candidate questionnaire items were developed from the individual themes, refined by piloting, cognitive interviews and a linguistic translatability assessment.

**Results:**

Thirty-six interviews were conducted to saturation with participants with GCA from the UK (25) and Australia (11). Mean age was 74 years, 23 (63.9%) were female, 13 (36.1%) had visual loss and 5 (13.9%) had LVV-GCA. Thirty-nine individual themes within five domains were identified: physical symptoms; activity of daily living and function; participation; psychological impact; and impact on sense of self and perception of health. Sixty-nine candidate items were developed from individual themes; piloting and refinement resulted in a 40-item draft questionnaire.

**Conclusion:**

This international qualitative study underpins the development of candidate items for a disease-specific PROM for GCA. The draft questionnaire is now ready for psychometric testing.

##  

**Rheumatology key messages**

- Health-related quality of life is significantly impacted in people with giant cell arteritis.
- Patients with GCA from the UK and Australia were interviewed about quality of life.
- Patient themes of importance have underpinned the development of a PROM for GCA.

## Introduction

GCA is the most common form of large vessel vasculitis (LVV) and typically presents with cranial ischaemic symptoms including headache, scalp tenderness and jaw claudication due to inflammation of the blood vessels of the head and neck [1]. Extracranial LVV-GCA, affecting the aorta and its main branches without cranial involvement, is also part of the spectrum of disease [2]. GCA presents over the age of 50, mainly in females. Seven out of 10 000 women aged 70–79 develop GCA every year [1]. GCA is a medical emergency due to the risk of blindness in 20% [3, 4]. Prompt treatment with high dose glucocorticoids (GCs) is needed to protect sight [5, 6] and has been the mainstay of treatment for over 60 years. Adverse effects are common including psychological symptoms, cardiovascular disease, osteoporosis and diabetes [7–9].

GCA and its treatment impact on people’s lives because of symptoms, adverse effects of GCs and disruption to normal life [10]. In a clinician-designed survey, people with GCA have previously ranked ‘losing sight in both eyes permanently’, ‘having intense or severe pain’ and ‘feeling weak, tired or exhausted’ as important quality of life domains [11].

Advances in knowledge about the pathological mechanisms involved in GCA have led to an explosion of interest in newer targeted therapies [12]. The anti-IL-6 inhibitor tocilizumab is effective [13] and improves health-related quality of life (HRQoL) [14]. Randomized controlled trials of novel therapies should assess efficacy based on outcomes of importance to both clinicians and patients. The patient perspective captured by validated patient reported outcome (PRO) measures provides valuable insights into the patient condition, which are not always captured by clinician-reported assessment tools [15].

The Outcome Measures in Rheumatology (OMERACT) Large Vessel Vasculitis Working Group have identified the need for a disease-specific patient-reported outcome measure (PROM) for GCA [16]. Generic PROMs, which can be used across a range of different diseases, may not always involve content specific enough for use in GCA. Short-Form-36 (SF-36) [17] scores in GCA studies do not always correlate with visual loss or systemic complications, potentially indicating a lack of sensitivity to differentiate between clinically important groups [18, 19]. Using both generic and disease-specific PROs should ensure accurate assessment of all outcomes of importance to patients with GCA [20]. Guidance from the US Food and Drug Administration (FDA) on the development of patient reported outcomes highlights the need for a patient involvement at each stage of development of the PROM [21].

The aim of this study is to explore the impact of GCA and its treatment on people’s health-related quality of life (HRQoL) from the patient perspective. HRQoL is multidimensional and includes physical, psychological and social functioning. These data will underpin the development of a disease-specific PROM for GCA.

## Methods

### Study management

Members of the study steering committee included patient research partners, qualitative researchers, methodologists and clinicians with an interest in GCA from the UK and Australia. As per FDA guidance [21], a conceptual framework was developed based on steering committee input and literature review to describe potential themes of interest; this was refined throughout the study based on interview content. An interview topic guide was also developed to include prompts and cues. The project was discussed in a Vasculitis Special Interest Group at an OMERACT conference. Ethical approval was obtained in the UK (South Central—Oxford B Research Ethics Committee; REC reference: 16/SC/0697, IRAS project ID: 217748) and Australia (Central Adelaide Local health Network; HREC Ref: HREC/17/TQEH/275 and CALHN Ref: Q20170906). All participants provided informed consent.

### Participants

Participants were recruited from two rheumatology and one ophthalmology clinic in the UK and Australia. Participants were made aware of the study by their usual clinical team. Interested participants were then sent an information sheet by the non-clinical research team; after reading this, participants could either decline the study or take part as they wished. Inclusion criteria included the following: a definite diagnosis of GCA confirmed by at least one diagnostic test: temporal artery biopsy, temporal artery ultrasound, CT angiogram or PET scan; age 18 or over; sufficient English language skills to participate in the interview; and the capacity to provide informed consent. An *a priori* purposive sampling framework was developed by the study steering committee to ensure a broad sample of participants with different demographic characteristics (age and sex), disease features (cranial, LVV and visual involvement), disease duration (less than, *vs* greater than 1 year) and current disease activity. A participant information sheet also captured data on education level and work. A clinical case report form collected data on presenting features, treatments used and diagnostic tests that aided completion of the purposive sampling grid as recruitment progressed.

### In-depth qualitative interviews

Informed consent was obtained prior to each semi-structured patient interview. Interviews, performed by experienced qualitative researchers in the UK (C.A.) and Australia (E.H.) were recorded, transcribed and anonymized. Initial interviews were used to determine the breadth of topics of importance to patients with GCA in relation to the disease and its treatment, and its impact on HRQoL. Australian transcripts were sent securely to the UK. All study transcripts were then organized by a qualitative researcher (C.A.) within one NVivo database prior to analysis. Inductive analysis was used, in conjunction with the conceptual framework for the PRO [21]. Initially, qualitative data were coded by reading the anonymized transcript multiple times and identifying important topics, including quotes and short phrases by C.A. and J.R. Individual themes were identified and given a descriptive label. In the second phase, the themes from the transcript were reduced and refined (excluding duplications and clarifying the meaning of each label) by C.A., J.R., J.D. and patient partner A.B. Individual themes were then grouped into overarching domains related to development of a PROM for GCA and its treatment.

### Candidate item development

A long list of candidate items was developed based on the individual themes and patient descriptions by C.A., J.R., J.D. and A.B. Items were added or removed or refined to improve the readability of each item and reduce overlap between items. Piloting and further amendment was conducted in an iterative way by steering committee patient partners.

### Cognitive interviews and linguistic evaluation

Serial cognitive interviews were conducted with patients in the UK and Australia. Cognitive interviewing involves interviewers asking survey respondents to think out loud as they read through a questionnaire to assess understanding of each item from the respondent’s perspective [22]. In this way, ambiguous or confusing items were amended or removed.

While cognitive interviews proceeded, the development of the PROM was critically reviewed at a Vasculitis Special Interest Group meeting at OMERACT 2018 [23]. In parallel, a face validity and linguistic assessment of the original English source text of the long list of candidate items was independently performed in accordance with current industry standards and guidance from the FDA by a specialist company (RWS Life Sciences, Buckinghamshire, England) (see Supplement 3, available at *Rheumatology* online).

## Results

Thirty-six interviews were conducted in two rheumatology and one ophthalmology site in Australia (11 interviews) and the UK (25 interviews). Twenty-eight interviews were via telephone and eight were face-to-face. Mean duration of interviews was 33.1 min (range 18–71 min). Demographic and disease features are shown in Table 1. All participants had confirmatory diagnostic tests for GCA: 31 (86%) had cranial disease, five (14%) had large vessel vasculitis disease and 13 (36%) had visual involvement. Mean age of participants was 74, and nine (36%) were male.

**Table 1:** Demographics and clinical features

	**UK** **(*n* = 25)**	**Australia** **(*n* = 11)**	**Total** **(*n* = 36)**
Demographics			
Sex, *n* (%)			
Male	9 (36.0)	4 (30.8)	13 (36.1)
Female	16 (64.0)	7 (69.2)	23 (63.9)
Age			
<70 years, *n* (%)	5 (20.0)	4 (36.4)	9 (25.0)
≥70 years, *n* (%)	20 (80.0)	7 (63.6)	27 (75.0)
Mean, years	75	73	74
Highest educational level, *n* (%)			
College/university	8 (32.0)	4 (36.4)	12 (33.3)
High school	6 (24.0)	4 (36.4)	10 (27.8)
Vocational/ employment	5 (20.0)	2 (18.1)	7 (19.4)
Employment, *n* (%)			
Retired	23 (92.0)	9 (82.0)	32 (88.9)
Employed with income	1 (4.0)	2 (18.1)	3 (8.33)
Disease features			
Diagnostic test, *n* (%)			
Biopsy	21 (84.0)	10 (90.9)	31 (86.1)
Temporal artery ultrasound	5 (20.0)	0 0	5 (13.9)
CTA	2 (8.0)	0 0	2 (5.6)
PET	2 (8.0)	1 (9.1)	3 (8.3)
Time from diagnosis, *n* (%)			
<1 year	13 (52.0)	6 (54.5)	19 (52.8)
≥1 year	12 (48.0)	5 (45.5)	17 (47.2)
Disease Active, *n* (%)			
Yes	11 (44.0)	6 (54.5)	15 (48.4)
No	14 (56.0)	5 (45.5)	16 (51.6)
Flare <1 year, *n* (%)			
Yes	13 (52.0)	5 (45.5)	16 (51.6
No	10 (40.0)	5 (45.5)	13 (41.9)
Never	2 (8.0)	1 (9.1)	2 (6.5)
Visual loss, *n* (%)			
Yes	10 (40.0)	3 (27.3)	13 (36.1)
No	15 (60.0)	8 (72.7)	23 (63.9)
PMR, *n* (%)			
Yes	11 (44.0)	5 (45.5)	16 (44.4)
No	14 (56.0)	6 (54.5)	20 (55.6)
ESR ≥50 or CRP ≥10, *n* (%)			
Yes	24 (96.0)	10 (90.9)	34 (94.4)
No	1 (4.0)	1 (9.1)	2 (5.6)
Additional immunosup- pressants, *n* (%)			
Methotrexate, lefluno mide or azathioprine	2 (8.0)	6 (54.5)	6 (16.7)
Tocilizumab or Sirukumab	—	3 (27.3)	3 (8.33)

CTA:CT angiogram; PET: Positron emission tomography.

Analysis of interview transcripts identified 111 individual themes. These were then refined through a process of discussion, amalgamation and refinement resulting in 39 individual themes within five overarching domains relevant to the development of a PROM including: physical symptoms; activities of daily living and function; participation; psychological impact; and impact on sense of self and perception of health. The saturation table of identified themes across the 36 interviews is shown in Supplementary Table S1, available at *Rheumatology* online. This demonstrates the final hierarchical coding structure (including individual themes and overarching domains), the number of references to each theme for each individual interview transcript, and the distribution of references across the dataset. The saturation table therefore demonstrates both the structure of the coding, or coding-tree, and the data-saturation across the dataset (i.e. no new themes were emerging with each interview).

Other themes of importance to participants included getting a diagnosis of GCA and patterns of active disease/flares. These themes are reported briefly below but would not be included within the scope of a PROM (which is used to assess patients’ views of their current health status).

### Domain 1: physical symptoms

The classical symptoms of GCA, including temporal headache and scalp tenderness, were described by participants. Descriptions varied, however, from mild symptoms, e.g. sensitivity not pain, twinges of headache, aching in the bones of the face and soreness of the scalp, through to severe, e.g. sharp knife-like pain, radiating pain, the sensation of a steel cap being screwed tightly on the head or the skull being in an ‘ice-bucket’.

*I couldn't stand the water on top of my head and I certainly couldn’t put a comb in my hair.* (UK 81-year-old male.)

Pain in the ear and neck were commonly reported as part of the headache. Four patients did not describe headaches or scalp tenderness, including 2 of the 5 patients with LVV-GCA. Pain in the jaw was described by over half the participants, particularly in relation to eating, chewing and even speaking and cleaning teeth.

Different patterns of eye involvement were described.

*It was a bit like watching a very cheap DVD on the television when it all goes crackle, crackle, crackle and breaks up;* *that’s exactly how my vision was.* (UK 74-year-old male.)

Participants described clouding, blurring or fuzziness of vision, the sensation of a net being thrown over the eye, losing patches of vision, double vision, feeling out of focus, and black and white vision (i.e. loss of colour). Some participants described sudden loss of vision, for example waking from sleep with no vision in one eye. The majority of participants with visual loss (8/15 patients) had some preceding non-visual symptoms in retrospect, for example muscle aches and pains, sweats, headaches and jaw pain, although the significance of these were only appreciated by the participants after diagnosis.

Musculoskeletal symptoms including joint pain and/or muscle stiffness and aching were commonly experienced particularly in the neck, shoulders, hips and legs in keeping with the known association with polymyalgia rheumatica. Systemic symptoms such as night sweats, lethargy and flu-like symptoms were also frequently reported. Other symptoms described included dizziness, weakness and unsteadiness, potentially related to vertebral involvement in those participants. Chest and abdominal discomfort were reported by participants with LVV, but also as a later feature attributed by patients as being related to glucocorticoid adverse effects. Change in appearance including weight and appetite gain were also linked to glucocorticoids by participants. Overall there were 11 individual themes identified (see Table 2).

**Table 2:** Physical symptoms of GCA in relation to disease and its treatment

Domain 1: physical symptoms
A. Headache and scalp sensitivity
* I had this radiating head, like horns coming out of the side of my head and lumps at the temples*. (AU 71-year-old female)
* I had terrible headaches;* *it was like somebody putting a steel cap and screwing it tight.* (UK 72-year-old female)
B. Ear and throat pain
* My ears were sore. Deep within the ears was pain, a fairly constant headache.* (AU 79-year-old male)
C. Jaw pain and stiffness, chewing and eating
* I could not chew and I couldn’t use my jaw, it hurt to talk, it hurt to move it.* (UK 75-year-old female)
* It was really, really difficult to eat. All I could have was soups, liquid foods.* (UK 64-year-old female)
D. Weight and appetite change
* I didn’t feel very well, which is unusual for me, and no energy and no appetite.* (UK 79-year-old female)
* I dropped weight within about* *2* *or* *3* *weeks;* *it just kept getting worse*. (AU 66-year-old female)
* I was getting up at 3 a.m. because I was so hungry. They* [steroids] *did make me very, very hungry.* (UK 72-year-old male)
E. Change in appearance due to treatment or disease
* My appearance has been a big thing to me, and I can't wear any of my clothes.* *I've got this huge stomach and skinny arms that you get with long steroid use.* (AU 71-year-old female)
F. Skin and hair symptoms
* But as the steroids take hold, if I brushed against a chair, the skin would lift off*. (AU 79-year-old male)
G. Dizziness, unsteadiness, weakness
* They didn’t like leaving me, cos I was very dizzy and falling around and losing my balance.* (UK 83-year-old female)
* I’m a bit weaker. I lack energy, mental and physical, compared with how I was*. (AU 79-year-old male)
H. Visual symptoms
* Double vision, out of focus, feeling dizzy;* *it was very frightening*. (AU 71-year-old female)
* In the bottom right hand corner of the eye as though someone was throwing a net over the eye*. (AU 85-year-old male)
* I woke up and realised that I’d lost, not all the sight, but part of the sight of my right eye*. (UK 79-year-old female)
* Well it started with my left eye going into a negative mode… almost black and white.* (UK 82-year-old male)
I. Joint and muscle symptoms
* I had so much stiffness in my joints and muscles and pain—it was unbelievable. I ache all across my shoulders, my back, and not only my hips but down my legs*. (AU 71-year-old female)
J. Systemic problems, lethargy, sweats and flu-like symptoms
* I did go through the terrible sweats in the night. I was getting up, changing my clothes three times.* (UK 72-year-old female)
* I felt like hell getting out of bed. My shoulders ached. My head ached and I just felt fluey.* (UK 72-year-old male)
K. Breathing problems, chest or gastric pain or discomfort
* In the night I had strange pains in the middle of my chest for about an hour radiating to the back.* (AU 71-year-old female)
* There's a bloating feeling around the stomach, lungs, chest area.* (AU 85-year-old male)

Quotes supporting the ‘Physical symptoms’ domain and 11 subthemes.

### Domain 2: activities of daily living and function

Participants reported that upper and lower limbs were affected, resulting in difficulties with lifting heavy objects, particularly above the shoulders, and problems with balance and personal mobility. See Table 3 for the seven individual themes identified and quotes.

**Table 3:** Activities of daily living and function (domain 2) and participation (domain 3**)**


Domain 2: activities of daily living and function
* *A. Upper limb arms/shoulders and hands
* Um, so I just couldn’t do anything, couldn’t raise my arms, couldn’t open the garage door, and just to change the gears on the car… it was painful…* (UK 79-year-old female)
* *B. Lower limb function and knees
* I just could hardly lift my legs. I couldn't get into a car. I did, but it was hell on Earth.* (AU 80-year-old female)
* Socks are a necessary bugbear. I can't stand up and balance. I've got to sit down*. (AU 85-year-old male)
* *C. Visual function
* I’d decide to make a coffee and then I’d pour the milk over the work surface instead of the cup.* (UK 72-year-old male)
* To read newspaper print with 12 inches or 15 inches? No way.* (AU 85-year-old male)
* *D. Essential household
* And even to cook a meal from scratch was too much.* (UK 64-year-old female)
* I used to do all my housework and everything like that. I find it very hard to vacuum now*. (AU 75-year-old female)
* I can't stand up. I haven’t got the strength to put my hands up, to hang the washing on the line*. (AU 75-year-old female)
* *E. Shopping
* Just lifting things, you know, like carrying the shopping or moving the hoover, anything heavy I do, I find my arms ache*. (UK 69-year-old female)
* *F. Walking and personal mobility
* At some stages I couldn't walk very far. I could only get out of bed and have a wheelchair and get to the bathroom and then back again.* (AU 80-year-old female)
* *G. Driving
* At night-time anything coming in from the side you don't see a thing.* (AU 85-year-old male)
* I saw three lorries coming towards me, and I couldn’t work out where the side of the road was*. (UK 67-year-old female)
Domain 3: participation
* *A. Family and caring roles
* It's affecting everything. My relationship. I've been married 48 years. Sometimes he'll say, let's go out and I just don't feel like it.* (AU 66-year-old female)
* I was quite poorly at Christmas, so the family took over, because I’ve always done Christmas here and I just literally gave in and said, ‘I’m sorry, but I can’t cope with it’*. (UK 82-year-old female)
* *B. Hobbies at home
* We’ve got a large garden; it’s always been my love and I haven’t been able to do any of that either.* (UK 79-year-old female)
* I can’t do knitting anymore, crochet, embroidery, crosswords,* *cos* *it’s very strenuous on my eyes*. (UK 78-year-old female)
* *C. Hobbies physical
* I felt I had to get out on my bike. I went off for a short run on the flat, and I could hardly get home.* (AU 79-year-old male)
* *D. Hobbies social
* If a phone rings, sometimes I'll say, oh no I don't want to, don't feel like that.* (AU 83-year-old female)
* I’ve dropped the things that I’ve enjoyed, tai chi and, and WI* [Womens’ Institute]. (UK 79-year-old female)
* *E. Work paid and voluntary
* I would like to go back to work, but I can’t, cos I ain’t got the strength*. (UK 83-year-old-female)
* I usually clean the church and set it up for Mass. I just can't, I can't lift anything.* (AU 75-year-old female)

*I haven't got the energy and I can't get my hands above my head to hang out the washing.* (AU 80-year-old female.)

Essential household tasks, and personal care such as dressing could be affected. Outside the house, there were some reports of difficulty with walking and shopping. In those with visual impairment, difficulties were experienced with reading and functioning in the home and impact on driving.

### Domain 3: participation

Functional limitations from GCA affected participants’ performance in their usual family caring roles, including grandparenting and other personal relationships. See Table 3 for five individual themes identified and further quotes.

*They used to call me Super Nan, but they can’t call me that now*. (UK 79-year-old female.)

An impact on hobbies was reported, due to visual impairment or lack of physical strength, at home and during sporting activities.

*When I played tennis I'd have to take a little stool out and, at the end of each swap over, sit down.* (AU 56-year-old female.)

Three of the participants reported they were currently employed. GCA and its treatment was reported as impacting work, and voluntary work roles, due to general lack of strength and limitations due to visual impairment (e.g. when performing tasks on computers).

*I was working full time. I hadn't retired. I was finding it very hard;* *even at work I had blurry vision.* (AU 66-year-old female.)

### Domain 4: psychological impact

Most participants described the emotional impact of GCA or its treatment; some participants also felt anger in relation to the process of getting the diagnosis. Participants described coping with daily fatigue, brain fog and problems with concentration.

*I was a bit shocked, easily agitated and aggravated.* (AU 79-year-old male.)

Irritability, low mood, depression and despair were all reported. Some of this was attributed to the disease itself, feeling unwell and not being able to participate as usual, and some was related to glucocorticoid use, particularly in the earlier stages involving high dosages. Glucocorticoids could also affect sleep quality, which worsened mental health and could lead to change in personality.

*I was taking 60 milligrams a day and I was hyper, hyper, hyper, slept for about 3 h at night.* (UK 72-year-old female.)

People felt increased sensitivity to noise and stress and in some cases reported choosing to socially isolate, to feel safe. Some had a fear of the future including fear of disease relapse, particularly in relation to visual loss. See Table 4 for the 10 individual themes identified and quotes.

**Table 4:** Psychological impact (domain 4) and impact on sense of self and perception of health (domain 5)


Domain 4: psychological impact
* *A. Anger, frustration, irritability
* Change in the character; my wife said I was a little bit irritable.* (UK 82-year-old male)
* *B. Brain fog and concentration
* It has affected my brain and I cannot think straight.* (AU 71-year-old female)
* *C. Coping and getting through the day
* I couldn’t cope, I was so tired, so exhausted and everything was painful.* (UK 64-year-old female)
* *D. Energy tiredness and fatigue
* I just can't understand why I'm so tired all the time and have got no energy.* (AU 75-year-old female)
* *E. Guilt
* I'm disappointed in myself, I suppose, that I'm not pulling my weight.* (AU 83-year-old female)
* *F. Low mood, depression, despair
* The depression is more like a flatness…* *some days I can feel completely flat, like I have no emotion*… (AU 71-year-old female)
* *G. Sensitivity to noise
* It’s not just noise, it almost vibrates the head, like having an empty bucket and it vibrates and throbs*. (UK 75-year-old female)
* *H. Sleep problems
* I can't sleep, and I'm up and about and I'll go and do the ironing at* *2* *o'clock in the morning*. (AU 80-year-old female)
* *I. Social isolation
* I feel like I'm not as social as I used to be. I just don't want to.* (AU 66-year-old female)
* I feel safer at home than being out in the public;* *I have dropped quite a few things I normally do.* (UK 79-year-old female)
* *J. Worry, anxiety, stress, fear of the future
* I'm much more nervous than I normally am and can't necessarily control that level of nerves*. (AU 62-year-old female)
* It was always in the back of my mind, oh my god, will this happen again?* (UK 72-year-old male)
Domain 5: impact on sense of self and perception of health
* *A. General health status, feeling normal, feeling ill, feeling older, ‘not like me’
* I’ve got a little bit of extra weight, which again is unlike me*. (AU 79-year-old male)
* *B. Heightened awareness
* And if I get a headache, oh god I hope this is not coming back again.* (UK 72-year-old male)
* *C. Independence and feeling in control *vs* needing support
* I don’t feel as if I can do anything to help myself when a relapse comes, which is always frustrating in itself because I think we like to have control of things.* (AU 62-year-old female)
* All I was worried about was losing me independence to be honest.* (UK 72-year-old male)
* *D. Source of others worries and concerns
* It's made my wife very, very nervous and it's unusual for her.* (UK 81-year-old male)
* Even now my daughter will say,* *‘um do you think you ought to do that?’* *I think she monitors things.* (UK 64-year-old female)
* *E. Travel long distance from home holiday or visiting
* Oh, we don’t get away on holiday now.* (UK 86-year-old female)
* *F. Treatment taking time and effort
* I don't know how older people manage quite honestly with everything—with their tablet taking*… (AU 71-year-old female)

### Domain 5: impact on sense of self and perception of health

Participants described heightened health-related awareness and vigilance in themselves and others, including a general impact on how they saw themselves, in terms of their health and capabilities. They felt older, less active and less confident. They did not feel ‘normal for them’.

*I’d like to think it’s all be back to normal eventually… but by then I shall forget what normal was.* (UK 78-year-old female.)

GCA was felt to dominate life for some people, with treatment and monitoring requiring thought and effort. Some participants reported losing independence and confidence to travel any distance from home. See Table 4 for the six individual themes identified and quotes.

*This has taken years, and I have been up and down. I've had about seven relapses of varying severity.* (AU 62-year-old female.)

### Patient experience of getting a diagnosis of GCA and increases in disease activity (disease flares)

Interview participants were keen to discuss the process of getting a diagnosis of GCA and their experiences of having an increase in disease activity (known as flares of disease). These themes would not be included in the development of a PRO (which measures partients’ views of their current health status) but could be the subject of future investigation.

Participants were often shocked at the diagnosis of GCA, particularly in terms of potential visual loss if not treated. Some participants reflected that in retrospect they had dismissed non-specific symptoms at home. Others had sought help but there had been a delay in diagnosis, particularly if symptoms deviated from the ‘classical’ cranial symptoms. Some people expressed anger over this, and suggestions were made about increasing education for the general population and medical profession. See Table 5.

**Table 5:** Patient experience of getting a diagnosis of GCA and increases in disease activity (disease flares)


Patient experiences of receiving a diagnosis of GCA
* I’d had these headaches a good* *18* *months. And I’d almost left it too late, because the doctor was, she said* *‘Another week or two, you’d have either have gone blind, or had a massive stroke’. Where I’d left it so long, but you, you don’t go to the doctor for a headache do you?* (UK 83-year-old female)
* It takes ages for them to find* [the diagnosis]*. It took 3 months for them to decide. And then, of course, when they decided I needed the steroids, then they told me, you have to start the steroids immediately now, because you could go blind.* (UK 75-year-old female)
Patient perceptions of disease state
A. Being stable (‘in remission’)
* Touch wood it seems to be alright and all the blood tests I had since show no recurrence of giant cell arteritis and all the other levels seem to be fine.* (UK 73 year old male)
* Well I'd actually say it's more than stable, I still think it's improving. So hopefully next week I should be dropping down to six milligrams. So each month I'm dropping down a milligram.* (AU 56-year-old female)
B. Increase in disease activity (‘disease flares’)
* *When you had the flare what symptoms did you experience? * Well… my vision went. No warning at all.* * *So you didn't have any neck and jaw pain? * Not on the second time.* (UK 80-year-old male)
* So I said my neck and my shoulders and the back of my head is bad again. The front wasn’t bad and the jaw was alright.* *She did a blood test just to be on the safe side and it was up.* (UK 83-year-old female)

*I was shocked when the doctor—the ophthalmologist said to me, you’ve got this—he said you’re within a whisker of going blind in one eye or both. Now, that thing is just striking like lightning around the community, because nobody knows about it. There should be some way in the community of having the major symptoms* *publicized*. (AU 79-year-old male.)

People who had experienced disease flares/increase in disease activity often reported similar symptoms to those they had first presented. However, they were aware of the significance of their symptoms, and so sought help quickly and effectively. Often only a few symptoms developed before they sought help and in some cases this was visual disturbance alone. See Table 5.

*I knew it straightaway—I didn’t want to let it get too bad. I feel that although the head pain wasn’t excruciating it was there, definitely*. (UK 64-year-old female.)

Having stable disease (in remission) was described by patients in relation to the following triad of features: (i) having no symptoms of GCA, (ii) stable blood monitoring, and (iii) being on a successful glucocorticoid reduction regimen.

### Candidate item development

The 39 individual themes within the five PROM domains were recast into an initial list of 69 questionnaire candidate items (most individual themes resulted in more than one potential candidate item). An iterative process of refinement and reduction of items through patient partner review, three rounds of cognitive interviews in the UK (*n* = 8) and Australia (*n* = 9) and incorporation of the Translatability Assessment findings (see Supplement S3, available at *Rheumatology* online) was then completed by the group (J.R., J.D., C.A. and patient partner A.B.).

Items were rejected by participants due to ambiguity or difficulty with completion. The item ‘Feeling normal for me’ was rejected due to difficulties in completing repeatedly over time; ‘Being the source of other people’s worry’ was felt to be too complex and likely to be applied in different ways by different participants, and ‘Difficulty getting together with friends’ was felt to be too ambiguous as it could relate to physical or psychological difficulties. In terms of the structure of the questionnaire, participants suggested separating into two types of question: symptom severity and level of difficulty in terms of psychological impact and completing tasks. The recall period in the stem of each question was changed from 1 week to 7 days to make it clearer to answer. Using specific examples within questions was appreciated as useful by participants, e.g. listing a range of essential household tasks, and this was therefore adopted across a range of candidate questionnaire items. Using the word ‘concern’ rather than ‘worry’ was encouraged as a less loaded description. Australian cognitive interviews probed all aspects of the questionnaire but additionally raised issues with language used in some questions; for example the use of the word ‘hoover’ in Australia is considered quite an old-fashioned English term and so was changed to ‘vacuum’ in the final version. The seventh version and final long-form of the draft GCA PRO consisted of 40 candidate items. An abridged version of the items retained is shown is Supplement S2, available at *Rheumatology* online.

**Fig. 1:**
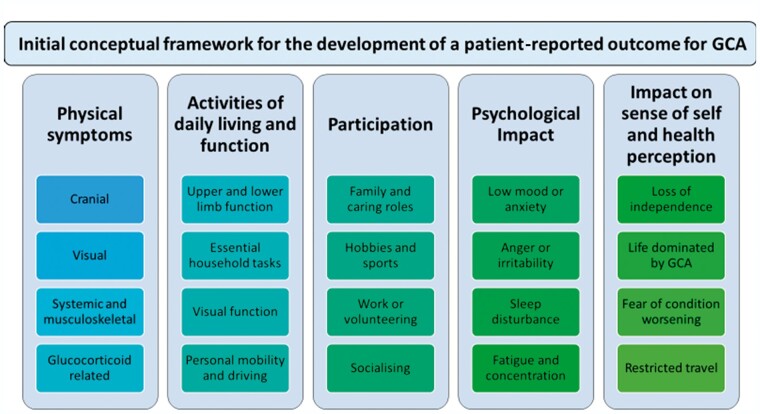
Initial conceptual framework for the development of a patient-reported outcome for GCA and its management

The initial conceptual framework was amended and refined during the qualitative work and the final version for this stage is shown in Fig. 1. Further item reduction and validation stages will be required before the final version of the conceptual framework and questionnaire is completed.

## Discussion

This international qualitative study examined themes of importance to people with GCA in relation to their disease, treatment and impact on HRQoL. This is the first study to develop a PROM for GCA with FDA-approved methods including patient involvement at every stage [21]. The PROM is based on five key domains: physical symptoms; activities of daily living and function; participation; psychological symptoms; and impact on sense of self and perception of health—resulting in 40 candidate questionnaire items.

The strengths of this study include the purposive sampling method used to ensure the inclusion of a wide range of participants likely representing the full range of impacts on HRQoL in GCA. Thus, participants were recruited from rheumatology and ophthalmology services in the UK and Australia, had a range of disease features including cranial disease, visual involvement and LVV-GCA, and different levels of disease activity and duration of disease. All participants had biopsy or imaging confirmed GCA.

One limitation is that this study was performed with only English-speaking people from the UK and Australia. Globally, GCA does have the highest incidence in populations of northern European ancestry [24] and limited evidence suggests that GCA is less common in non-Caucasian populations. It is possible that people from other countries may, however, report different themes of importance in relation to GCA. A linguistic evaluation has been performed, with adaptations made to the questionnaire items in order to ensure formal translations into a range of languages will be possible in the future.

Previous studies of HRQoL based in the UK [10] and the USA [11] have identified similar themes, including loss of normality, impact of physical symptoms—including from use of glucocorticoids—and concerns about visual loss, pain, weakness and fatigue [10, 11], which confirms the saliency of these findings. This, however, is the first study to use patient themes and wording to develop candidate questionnaire items using FDA-approved methods [21].

Some themes of importance to participants were identified in the data that would not be included in a PROM but could be important underpinning data for a future patient-related experience measure (PROMs) that examines patients’ experience of receiving care within their healthcare system [25]. In line with a 2007 meta-analysis reporting a mean delay to diagnosis of 9 weeks or longer [26], interview participants were very concerned about delays in diagnosis and lack of awareness of the disease. Participants’ perceptions of disease activity may inform the OMERACT Large Vessel Vasculitis Working Group’s ongoing work into defining disease state [23].

This project has completed the underpinning qualitative stages in the development of a disease-specific PROM for people with GCA. PROMs can be completed on paper, by post or electronically; this can facilitate remote follow-up and enhance patient-centred care, as well as being key outcomes of importance in clinical trials [27]. The next steps will include further development and validation of the 40-item draft questionnaire, using factor and Rasch analysis to determine scale structure (including reduction in number of items) and measurement properties in a large scale survey to complete the final GCA PROM.

*Funding:* Funding from the Above and Beyond Grant University Hospitals Bristol NHS Foundation Trust and QR Funding, University of the West of England, Bristol.

*Disclosure statement:* S.L.M. reports consultancy on behalf of her institution for Roche/Chugai, Sanofi, AbbVie and AstraZeneca, and received support from Roche to attend EULAR2019. S.L.M. is supported by the Leeds Biomedical Research Centre. The views expressed in this article are those of the authors and not necessarily those of the NIHR or the Department of Health and Social Care. No other authors have conflicts of interest to disclose in relation to this work.

## Data availability statement

Available upon request.

## Supplementary data

Supplementary data are available at *Rheumatology* online.

## Supplementary Material

keab076_supplementary_data
