# Supplementary material for: Patient perceptions of health-related quality of life in giant cell arteritis: international development of a disease-specific patient-reported outcome measure
Source: Rheumatology (Oxford). 2021 Feb 2;60(10):4671–80. doi: 10.1093/rheumatology/keab076 (PMC8487303; doi:10.1093/rheumatology/keab076)
Supplement: keab076_supplementary_data [file keab076_supplementary_data.zip › rhe-20-2769-File002.docx]

**Supplementary materials**

**S1. Saturation Table of themes and interviews**

**S2. Longlist of candidate questionnaire items.** Abridged items. The exact wording of the stem of each question, examples of symptoms and difficulties included within each item, and responses categories not included. Further validation required in large scale survey to determine scale structure and composition of items before can be used in clinical trials or practice.

| **SYMPTOMS Experience of..** | | **DIFFICULTIES with..** | | **DIFFICULTIES with..** | |
| --- | --- | --- | --- | --- | --- |
| 1 | Headaches | 14 | Raising arms | 28 | Feeling low |
| 2 | Jaw | 15 | Getting up from chair | 29 | Worried or anxious |
| 3 | Joints | 16 | Bending forward | 30 | Worried condition worsening |
| 4 | Sight | 17 | Using eyes | 31 | Socialising |
| 5 | Fatigue | 18 | Walking | 32 | Travel further from home |
| 6 | Chest pain | 19 | Routine tasks | 33 | Shopping |
| 7 | Dizziness | 20 | Getting out of bed | 34 | Getting together |
| 8 | Earache | 21 | Lifting | 35 | Life dominated |
| 9 | Sweating | 22 | Unsteady | 36 | Exercising |
| 10 | Stomach | 23 | Walking up stairs | 37 | Hobbies at home |
| 11 | Scalp tenderness | 24 | Appearance | 38 | Social activities |
| 12 | Skin | 25 | Angry or irritable | 39 | Responsibilities |
| 13 | Sleep | 26 | Concentrating | 40 | Local travel |
|  |  | 27 | Losing independence |  |  |

**S3 Description of Translatability Assessment performed by RWS Life Sciences for the GCA PRO project**

The objective was to assess the face (content) validity and translatability of the original English source text of the Giant Cell Arteritis (GCA)-PRO in accordance with current industry standards and guidance from the U.S. Food and Drug Administration (FDA).

# Face Validity and Translatability Assessments

## A. Methods

To assess the content validity of the original English source text, a face validity assessment was conducted by a survey research analyst and one quality assurance project manager who reviewed the English source text of the Giant Cell Arteritis (GCA)-PRO in order to determine which terms might cause issues during translation. Suggestions for alternative source items within the GCA-PRO were proposed and included for linguists’ consideration during the translatability assessment process.

To assess the translatability of the new source GCA-PRO a team of 10 linguists from 10 different countries reviewed the instrument to identify any concepts, phrases or components of the instrument which would be difficult to translate or appeared to be culturally-specific. The 10 countries represented are as follows: *China Simplified (Chinese); Finland (Finnish); France (French); Germany (German); Greece (Greek); India (Hindi); Arabic (Saudi Arabia); Singapore (Tamil); South Africa (Xhosa); Ukraine (Ukrainian)*

## B. Results

Following the face validity assessment, 10 linguists from 10 *languages* were given the source file of the Giant Cell Arteritis (GCA) PRO and were asked to evaluate the translatability of the instrument. Although the linguists found the Giant Cell Arteritis (GCA) PRO to be generally translatable, all 10 languages identified a small number of issues they thought might cause translation issues.

## C. Conclusion

The survey research analyst and quality assurance Project Manager have reviewed the results of the translatability assessments from the 10 linguists and made recommendations to the client for changes that could increase the translatability of the instrument across the 10 languages*.*

Examples of recommendations and changes made to the candidate questionnaire items:

Within the question related to “difficulty with lifting heavy objects”, the example “Heavy washing” was problematic in Hindi (India), and Arabic (Saudi Arabia), and was therefore removed as an example from the question.

Within the “Problems sleeping” question, “Getting to sleep” was difficult to translate into Arabic (Saudi Arabia) and was therefore changed to “falling asleep”.

In Greek (Greece) and Tamil (Singapore), “Pain” and “ache” are both translated the same and used interchangeably. In Tamil (Singapore): “Tiredness” and “fatigue” have same meaning. Although no change was made to these question, this was noted for future formal translation purposes.
