# Supplementary figures and images for: Patient perceptions of health-related quality of life in giant cell arteritis: international development of a disease-specific patient-reported outcome measure
Source: Rheumatology (Oxford). 2021 Feb 2;60(10):4671–80. doi: 10.1093/rheumatology/keab076 (PMC8487303; doi:10.1093/rheumatology/keab076)

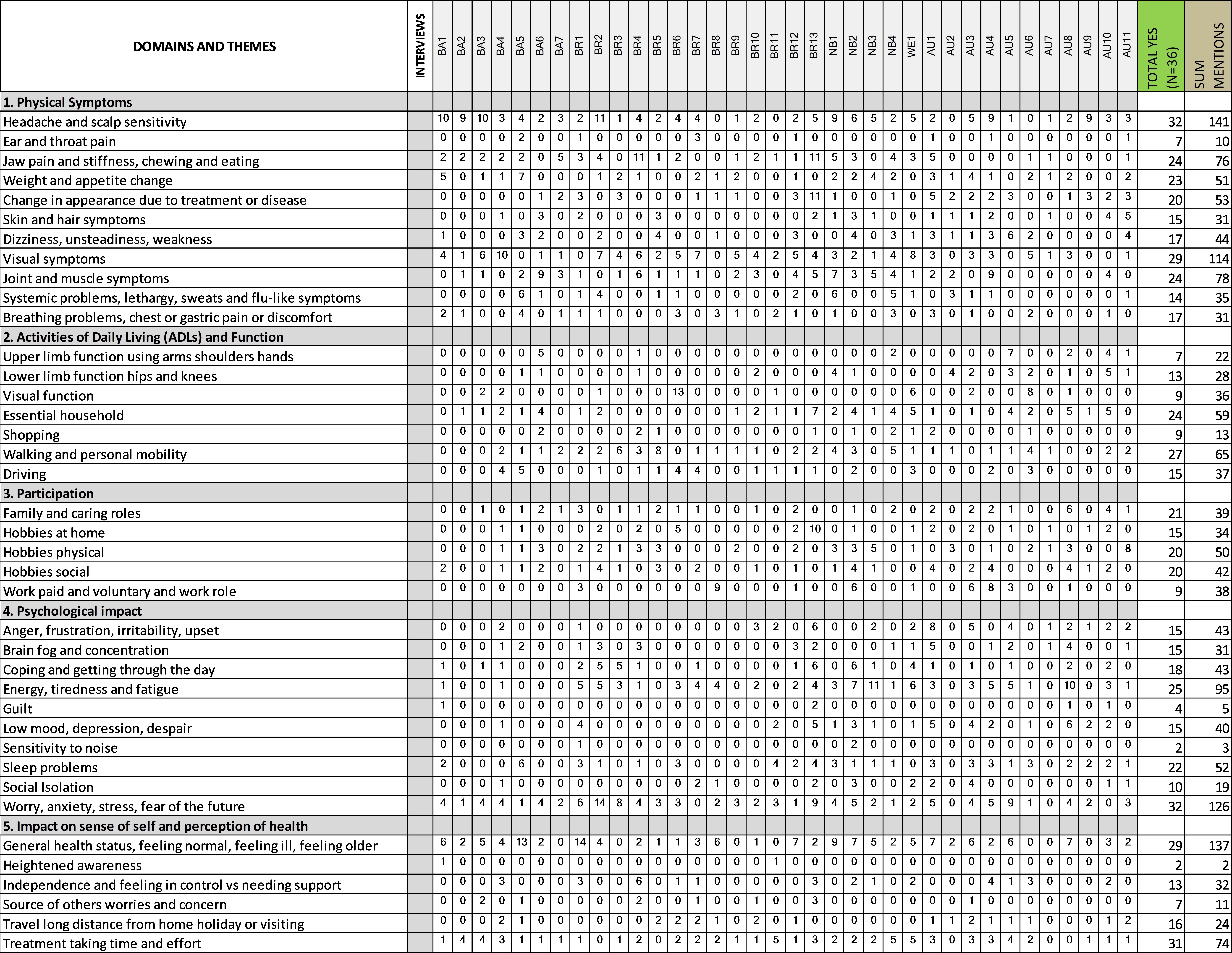

Supplement: keab076_supplementary_data [file keab076_supplementary_data.zip › rhe-20-2769-File005.png]
